# Systems approach to define humoral correlates of immunity to *Shigella*

**DOI:** 10.1016/j.celrep.2022.111216

**Published:** 2022-08-16

**Authors:** Biana Bernshtein, Esther Ndungo, Deniz Cizmeci, Peng Xu, Pavol Kováč, Meagan Kelly, Dilara Islam, Edward T. Ryan, Karen L. Kotloff, Marcela F. Pasetti, Galit Alter

**Affiliations:** 1 Ragon Institute of MGH, Harvard and MIT, Cambridge, MA, USA; 2 Department of Pediatrics, Center for Vaccine Development and Global Health, University of Maryland School of Medicine, Baltimore, MD, USA; 3 NIDDK, LBC, National Institutes of Health, Bethesda, MD, USA; 4 Division of Infectious Diseases, Massachusetts General Hospital, Boston, MA, USA; 5 Department of Medicine, Harvard Medical School, Boston, MA, USA

**Keywords:** antibodies, *Shigella*, Fc receptor, antibody functionality, human immunology, humoral immunity

## Abstract

*Shigella* infection is the second leading cause of death due to diarrheal disease in young children worldwide. With the rise of antibiotic resistance, initiatives to design and deploy a safe and effective *Shigella* vaccine are urgently needed. However, efforts to date have been hindered by the limited understanding of immunological correlates of protection against shigellosis. We applied systems serology to perform a comprehensive analysis of *Shigella-*specific antibody responses in sera obtained from volunteers before and after experimental infection with *S*. *flexneri* 2a in a series of controlled human challenge studies. Polysaccharide-specific antibody responses are infrequent prior to infection and evolve concomitantly with disease severity. In contrast, pre-existing antibody responses to type 3 secretion system proteins, particularly IpaB, consistently associate with clinical protection from disease. Linked to particular Fc-receptor binding patterns, IpaB-specific antibodies leverage neutrophils and monocytes, and complement and strongly associate with protective immunity. IpaB antibody-mediated functions improve with a subsequent rechallenge resulting in complete clinical protection. Collectively, our systems serological analyses indicate protein-specific functional correlates of immunity against *Shigella* in humans.

## Highlights

• Serological profiling of *Shigella* human challenge studies indicates protective markers
• Pre-existing IpaB-specific functional antibodies associate with less severe disease
• OPS immune responses post challenge are linked to less severe disease
• *Shigella* rechallenge boosts IpaB but not OPS functional antibody responses

## Graphical abstract

**Figure:**
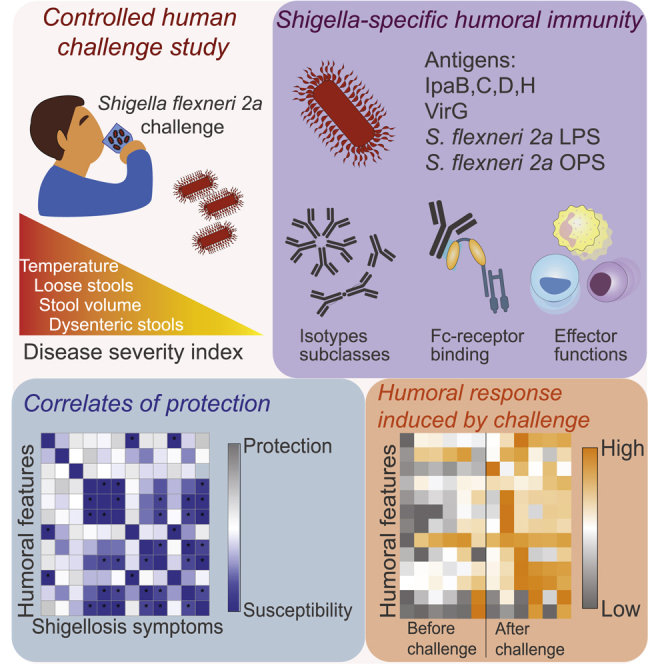

## Teaser

Bernshtein et al. profile antibody responses against *Shigella*, an enteropathogen that causes diarrhea and death in young children. System serology analysis of serum obtained in controlled human challenge studies show that high levels of pre-existing IpaB-specific antibodies and their Fc-receptor functions are associated with clinical protection against shigellosis.

## Introduction

*Shigella* infection is the second leading cause of death due to diarrheal disease in young children worldwide (Kotloff et al., 2013; Platts-Mills et al., 2015; Wang et al., 2016), primarily affecting low-income countries (Platts-Mills et al., 2015). Every year 200,000 people die of *Shigella* infection, more than 50,000 of these deaths occurring in children under 5 years of age (Platts-Mills et al., 2015). Moreover, in endemic regions, recurring *Shigella* infections cause lifelong disabilities including growth retardation and cognitive impairment (Kyu et al., 2018). Additionally, with the rise of drug-resistant strains, outbreaks of shigellosis are on the rise worldwide (Arvelo et al., 2009; Brian et al., 1993). Collectively, there is an urgent need for a vaccine to prevent *Shigella* infection (Barry et al., 2013; Levine et al., 2007). However, vaccine development has been hindered, in part due to our limited understanding of the correlates of immunity against *Shigella*.

Epidemiologic and animal model data both point to antibodies as key correlates of protection against *Shigella* (Arevalillo et al., 2017). Lipopolysaccharide (LPS)-specific (Cohen et al., 1988), IpaB-specific, and VirG-specific immunoglobulin G (IgG) (Shimanovich et al., 2017) have been associated with reduced risk of infection in humans. In the setting of natural exposure, early studies have shown an association between O-antigen-specific antibodies and protection against shigellosis (Cohen et al., 1988, 1991), strongly motivating the development of several O-specific polysaccharide (OPS)-based vaccine candidates (Mani et al., 2016). LPS-specific antibodies have been implicated in complement-mediated bactericidal activity (Micoli et al., 2021; Lin et al., 2016), and serum bactericidal activity has been explored as a predictor of protection from infection (Clarkson et al., 2020, 2021; Shimanovich et al., 2017) and as a functional marker of vaccination (Micoli et al., 2021; Sarker et al., 2021). In addition to the O antigen, antibodies to particular bacterial virulent factors have been implicated in antimicrobial activity by blocking cell invasion (Skoudy et al., 2000; Lafont et al., 2002). However, the precise combination of antigenic targets and antibody functions that confer immunity remains incompletely understood.

Beyond their ability to bind and block infection, antibodies are capable of deploying a large array of Fc-mediated functions, via the recruitment of the innate immune system, which contribute to host defense (Lu et al., 2017).The precise antigen-specific antibody functions that prevent *Shigella* infection are not known, and such knowledge could provide valuable insights to inform the development of a vaccine against this pathogen. Therefore, in this study, we leveraged a controlled human challenge model of experimental infection with virulent *S*. *flexneri* 2a (Kotloff et al., 1995a), the most abundantly isolated *Shigella* species worldwide (Kotloff et al., 1999), to objectively and comprehensively profile the functional humoral immune response to *Shigella* in subjects with well-defined *Shigella* exposure and clinical outcome. Antibodies against LPS of the two most prevalent *Shigella* strains, *S*. *flexneri* 2a and *S*. *sonnei*, *S*. *flexneri* 2a OPS, and virulence proteins IpaB, IpaC, IpaD, IpaH, and VirG were profiled.

As expected, robust, serotype-specific antibody responses evolved following *S*. *flexneri* 2a challenge. OPS-specific antibodies increased concomitantly to symptom severity, whereas pre-challenge IpaB-specific antibody levels and functions were inversely correlated with symptom severity. Moreover, IgG-mediated IpaB-specific Fc-effector profiles prior to infection were linked to protection, whereas post-infection IgA-mediated activity was associated with clinical protection, indicating distinct temporal antibody-associated protective mechanisms. These data point to a critical role for functional IpaB-specific Fc-effector humoral response that may be supported by additional LPS/OPS-specific antibody functions in the prevention of shigellosis.

## Results

### Profiling serum antibody responses to *S*. *flexneri* 2a infection

To begin to define the potential importance of qualitative differences in humoral immune responses to *Shigella*, we focused on a controlled human infection model (CHIM) in United States adults with tightly controlled exposure and systematic disease monitoring. This enabled a comprehensive analysis of antibody profiles that track with clinical protection against shigellosis. We analyzed a cohort of 27 healthy adult volunteers (cohort 1) that had been enrolled in a challenge study at the University of Maryland (Table S1). One month prior to challenge, 16 of the volunteers had been immunized orally with a live hybrid *Escherichia coli*-*S. flexneri* 2a vaccine strain, EcSf2a-2 (Kotloff et al., 1995a). Recipients of the vaccine or placebo were subsequently challenged with wild-type (WT) *S*. *flexneri* 2a. The vaccine was poorly immunogenic, with the overwhelming majority of recipients not developing measurable serum anti-LPS or anti-Ipa responses, resulting in only 27% efficacy (95% confidence limits −197, 82; p = 0.15) against challenge (Kotloff et al., 1995a). Principal component analysis (PCA) of pre-existing and post-challenge antibody profiles across individuals that had previously received the EcSf2a-2 or not showed limited differences of the *Shigella*-specific antibody response between the two groups (Figure S1A), consistent with the lack of vaccine efficacy. Additionally, no evidence of a unique memory expansion was noted in the vaccine recipients (Figure S1B, right panel). Moreover, no differences were noted in disease severity across the vaccinated and naive volunteers (Figure S1C), arguing that based on immunogenicity and antimicrobial responses, the combination of both groups of challenged volunteers was appropriate. Serological data from both groups were therefore combined and analyzed together, irrespective of whether the participants had received EcSf2a-2 or placebo prior to challenge with WT *S*. *flexneri* 2a.

**Figure 1:**
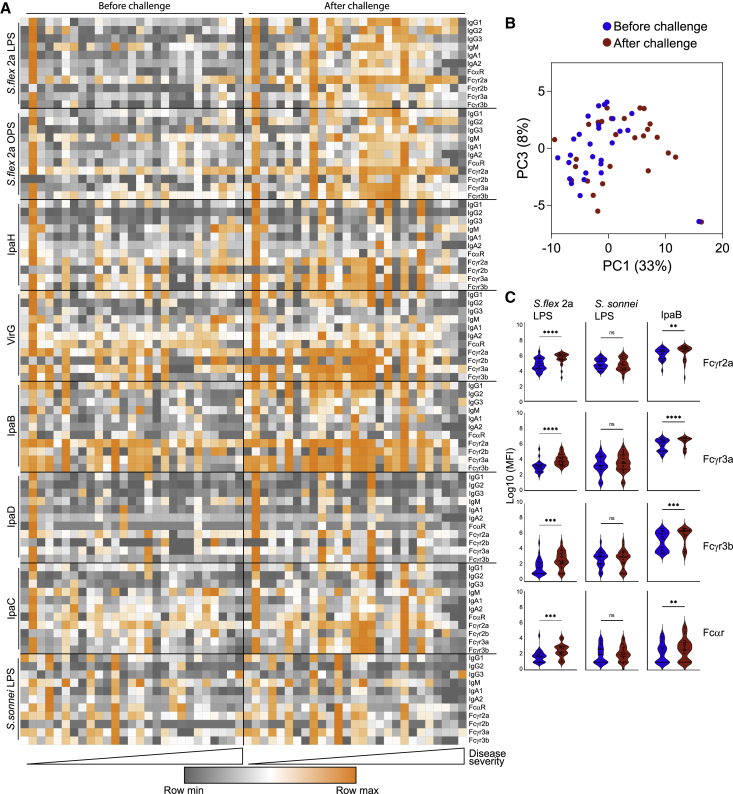
*S*. *flexneri* 2a challenge induces a robust and broad antibody response (A) Heatmap depicting antibody isotype and FcR binding before and after challenge with *S*. *flexneri* 2a (n = 27). (B) PCA of antibody profiles before and after *S*. *flexneri* 2a challenge (n = 27). (C) FcR binding of *S*. *flexneri* 2a LPS, IpaB, and *S*. *sonnei* LPS-specific antibodies measured by Luminex, before and after *S*. *flexneri* 2a challenge (n = 27) (^∗∗^p < 0.01, ^∗∗∗^p < 0.001, ^∗∗∗∗^p < 0.0001, Wilcoxon test; ns, not significant).

While the presence of pre-challenge IpaB- and VirG-specific IgG antibody titers was previously linked to reduced illness after *Shigella* challenge (Shimanovich et al., 2017), here we used systems serology to broadly profile and define the *Shigella*-specific functional humoral immune response to gain enhanced mechanistic insights into correlates of immunity. Specifically, we tested *Shigella*-specific antibody isotype, subclass, and Fc-receptor (FcR) binding profiles across IpaB, IpaC, IpaD, IpaH, VirG, *S*. *flexneri* 2a LPS and OPS, and *S*. *sonnei* LPS antigens (Figure S2). Low-level LPS- and OPS-specific IgG and IgA responses were detected in most volunteers prior to challenge (Figure 1A). Protein-specific pre-existing antibody levels varied, with low or sporadic IpaH, IpaD, and IpaC responses, but more robust and uniform responses against VirG and IpaB (Figure 1A). However, after challenge, IpaH-, VirG-, IpaB-, and IpaC-specific isotype, subclass, and FcR binding responses increased robustly, while responses to IpaD remained low (Figure 1A). PCA comparing the antibody profiles before and after challenge highlighted the clear shift in *Shigella*-specific humoral profiles along principal component 1 (PC1) driven by infection (Figure 1B). *S*. *flexneri* 2a challenge induced robust IpaB-specific FcR binding antibodies that were serotype specific, as *S*. *sonnei* LPS FcR binding antibodies did not increase after challenge (Figure 1C). Collectively, these data point to variable pre-existing polysaccharide and protein antigen immunity, both of which increased upon challenge.

### Pre-existing IpaB antibodies, but not OPS antibodies, track inversely with *S*. *flexneri* 2a disease severity

**Figure 2:**
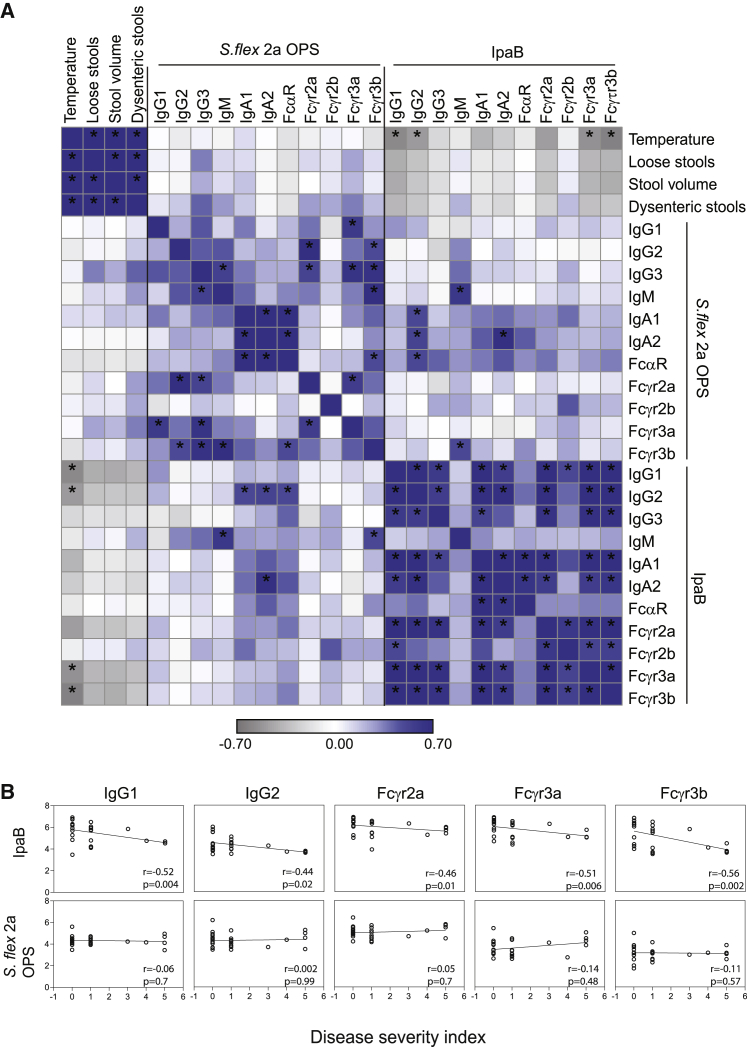
IpaB-specific antibody levels and FcR binding before *S*. *flexneri* 2a challenge negatively correlate with symptom severity (A) Heatmap of Spearman correlation between shigellosis symptoms and IpaB- and OPS-specific antibody profiles before *S*. *flexneri* 2a challenge. (n = 27). ^∗^Corrected p < 0.05. (B) Spearman correlation of disease severity index and selected antibody isotypes and FcR binding before *S*. *flexneri* 2a challenge (n = 27).

To define the specific antibody features and functions associated with protective immunity, we first examined the relationship between pre-existing *Shigella*-specific antibodies and symptoms of shigellosis (Figure S3). Given the collective data pointing to a critical role for OPS-specific IgG titers in protective immunity (Cohen et al., 2019), we initially examined the relationship between pre-existing OPS-specific responses and disease phenotypes. While both OPS and LPS were used for isotype and FcR binding analysis, OPS was used as the representative O antigen for further analysis and functional assays. No significant correlations were observed between all pre-challenge OPS-specific antibody features and shigellosis symptoms (Figure 2A). Conversely, pre-challenge IpaB-specific responses were strongly negatively associated with symptoms. Specifically, pre-challenge IpaB-specific IgG and IgA levels and Fcγ receptor binding negatively correlated with all measured shigellosis symptoms following challenge, including fever, number of loose stools, stool volume, and number of dysenteric stools (Figure 2A). Interestingly, baseline IpaB IgM and IpaB antibody binding to the inhibitory Fc receptor FcγR2b showed a weak positive correlation with shigellosis symptoms, and particularly with the number of dysenteric stools (r = 0.2, p > 0.05 for both). Moreover, pre-existing responses to OPS were weakly correlated with pre-existing responses to IpaB, suggesting that OPS and IpaB responses are induced or maintained independently.

To capture the overall relationship between individual antibody features and disease severity, a shigellosis disease severity index (DSI) was calculated for each challenged individual as previously described (Porter et al., 2018). In brief, body temperature and number of loose and dysenteric stools were scored separately and then combined to calculate DSI. The calculated DSI correlated well with the categorical 4-point disease index previously described for this cohort (Wahid et al., 2013; Shimanovich et al., 2017) (Figure S4). Pre-challenge IpaB-specific IgG1 and IgG2, as well as FcγR2a, FcγR3a, and FcγR3b binding, exhibited significant negative correlations with DSI, pointing to a potential role for these antibody responses in antimicrobial control (Figure 2B). Conversely, OPS-specific antibody levels and FcR binding pre-challenge in these samples did not correlate with DSI (Figure 2B). Thus, prior to challenge, IpaB, but not OPS-specific antibody responses, tracked inversely with symptom severity.

### Antibody functional correlates of *Shigella* protective immunity

**Figure 3:**
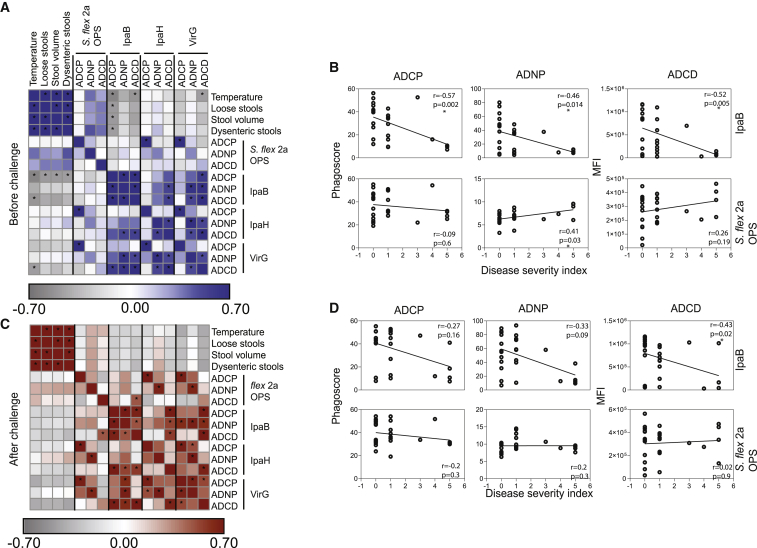
IpaB-specific antibodies mediate protective phagocytosis and complement deposition (A and C) Heatmap of Spearman correlation of shigellosis symptoms and OPS-, IpaB-, IpaH-, and VirG-specific antibody-mediated functions before (A) and after (C) *S*. *flexneri* 2a challenge. (n = 27). ^∗^Corrected p < 0.05. (B and D) Spearman correlation of disease severity index and OPS- or IpaB-specific antibody-mediated functions before (B) and after (D) *S*. *flexneri* 2a challenge (n = 27).

Given the striking differences in relationships between pre-existing OPS- and IpaB-specific antibody profiles with severity of shigellosis, we next aimed to define specific antibody effector functions that were selectively associated with protective immunity both before and after challenge. Analysis of pre-challenge OPS antibody functional profiles pointed to a positive correlation between antibody-dependent neutrophil phagocytosis (ADNP) and antibody-dependent complement deposition (ADCD), but not antibody-dependent cellular monocyte phagocytosis (ADCP), with disease symptoms (Figure 3A). However, only OPS ADNP had a significant positive correlation with disease severity (Figure 3B). Conversely, IpaB ADCP, ADCD, and to some extent ADNP pre-challenge levels negatively correlated with symptoms of shigellosis (Figure 3A), and all three exhibited significant negative associations with the severity index (Figure 3B). Pre-challenge VirG-specific ADCP and ADCD tracked inversely with symptoms of shigellosis such as number of dysenteric stools (r = −0.2 for both, p > 0.05), and VirG-specific ADCP and ADCD before challenge were robustly negatively correlated with disease severity (Figure S5), confirming the relevance of ADCP and ADCD as correlates of immunity, as similar functions were observed for IpaB (Figure 3A). Different from IpaB and VirG, IpaH antibodies were not associated with protection in our analysis; in fact, baseline IpaH-specific antibody functions were positively, but not significantly, correlated with disease symptoms (Figure 3A). Namely, IpaH-specific ADNP showed weak positive correlation with the number of dysenteric stools (r = 0.2, p > 0.05). Importantly, low correlation was observed between IpaB and OPS antibody functions before challenge (Figure 3A).

To further determine whether distinct functional humoral mechanisms emerged following challenge that would provide insights into the role of anamnestic protective responses, we also examined the relationship between post-challenge humoral functional profiles and symptoms (Figure 3C) and composite disease severity scores (Figure 3D). Specifically, post-challenge OPS-specific ADNP and ADCD were positively correlated with individual symptoms, whereas weak but negative correlations were largely observed for IpaB, IpaH, and VirG (Figure 3C).

In sum, IpaB-specific and, less prominently, VirG-specific ADCP and ADCD prior to challenge were negatively correlated with symptom severity; these results point to an important role in ADCP and ADCD mediated by protein-specific antibodies detected before challenge in protection against disease.

### Distinct pre- and post-challenge correlates point to distinct mechanisms of antibody action

**Figure 4:**
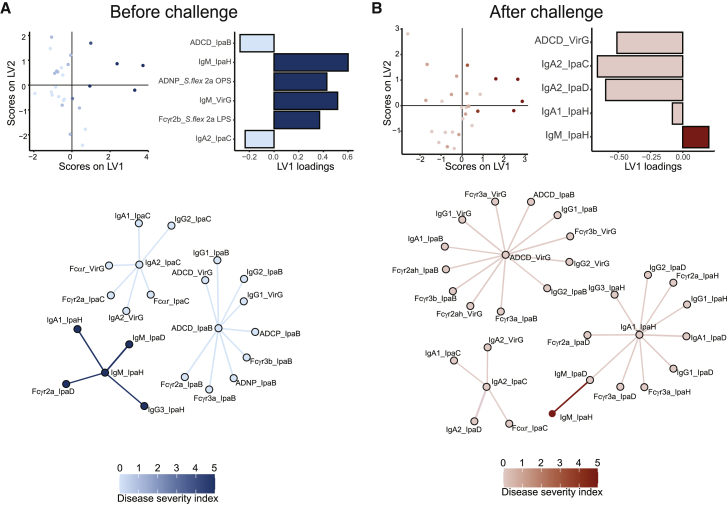
Multivariate analysis of the antibody response to *Shigella* challenge highlights IpaB antibody-mediated protection (A and B) LASSO-PLSR and correlation network of antibody features before (A) and after (B) challenge (n = 27).

Given the observed correlations of several *Shigella*-specific biophysical and functional antibody features determined before and after challenge with disease severity, we next aimed to define the minimal set of antibody features that could predict protection or that emerged as contributing to protective immunity against *Shigella*. A least absolute shrinkage and selection operator (LASSO) was used to downselect a set of minimal antibody features that differed most with disease severity (DSI score), first in the data collected prior to challenge (Figure 4A) and then in the data collected after the challenge (Figure 4B). Partial least-squares regression (PLSR) was used to visualize the data in both settings based on disease severity (Figures 4A and 4B). As few as 6 out of a total of 99 analyzed antibody features captured by systems serology pre-challenge were sufficient to separate individuals based on disease severity (Figure 4A). IpaB ADCD and IpaC-IgA responses were enriched in protected individuals prior to challenge. Conversely, OPS ADNP, LPS-specific FcγR2b-binding, and elevated IgM responses to both VirG and IpaH were enriched in individuals who ultimately developed severe disease. Importantly, no differences were observed in these biomarkers prior to challenge across the vaccinated and naive volunteers, arguing that the vaccine did not skew the response associated with protection (Figure S6A). These data point to pre-existing Ipa-specific class-switched complement-activating immunity, rather than LPS-targeted immune responses, as predictors of protection in this cohort.

To define a minimal set of biomarkers associated with protection, the LASSO algorithm eliminates co-correlated features and selects a minimal set of features that accounts for variation across disease severity. The co-correlates of the LASSO-selected features may help identify overall antibody profiles that are differentially enriched among individuals who resisted or experienced shigellosis. LASSO-feature correlation network analyses revealed three correlation networks before challenge (Figure 4A). First, IpaB-mediated complement deposition was correlated with additional IpaB-specific Fc-mediated functions (ADNP and ADCP) as well as IpaB IgG antibody subclass selection and FcR binding, all pointing to the importance of pre-existing IgG-class-switched and functional humoral immune responses. Additionally, VirG ADCD was correlated with IpaB ADCD, implying a potential collaboration between IpaB and VirG functions in protection. Furthermore, IpaC-IgA2 was likewise enriched among individuals with milder disease following challenge and was tightly linked to enhanced VirG IgA2 and Fcα-receptor (FcαR) binding in addition to IpaC IgA1, IgG2, and FcαR and FcγR binding in protected participants. This suggests an overall functional-IgG-driven IpaB/VirG and an IgA-dominated IpaC/VirG profile associated with protective immunity against *Shigella* (Figure 4B). Conversely, IpaH-specific IgM was linked to elevated IpaD-IgM and several IpaH features (i.e., IgG3 and IgA1), denoting a non-class-switched IpaH and IpaD as well as a class-switched IpaH immunity that tracked with disease susceptibility. Additionally, a PLSR model was built separately on the subset of 11 naive individuals without downselection of features using LASSO, owing to the limited size of the group (Figure S6). Nonetheless, pre-challenge features that were associated with protection in the naive group (Figure S6B) recapitulated those that were found for the naive and vaccinated merged cohort, including IpaB-specific functions, while IpaH-specific IgM was associated with severe disease.

Following challenge, the LASSO/PLSR required as few as five features to discriminate individuals who experienced mild and severe disease. A striking bias toward expanded complement-fixing VirG and IgA immunity to IpaC, IpaD, and IpaH was observed in individuals with milder or no disease. Interestingly, complement-fixing VirG-specific antibodies were expanded in protected naive individuals post challenge as well (Figure S6C). Conversely, a robust expansion of IpaH-IgM responses was observed in individuals who experienced severe disease (Figure 4B). This likely indicates that IpaH is a sensitive indicator of shigellosis; the *ipaH* gene is commonly used as a target for molecular diagnosis of *Shigella* in fecal samples (Liu et al., 2016). The co-correlates network revealed three large clusters of mixed class-switched IgA and IgG/FcR binding antibody responses across the Ipa and VirG antigens in individuals who experienced minimal disease. While LPS-specific responses were expanded following challenge (Figures 1A and 1C), robust pre-existing and post-challenge expanded pan-protein IgA and IgG/Fc immunity represented stronger biomarkers of protective immunity in this population.

### Comparative analysis of polysaccharide and protein-specific antibody function

**Figure 5:**
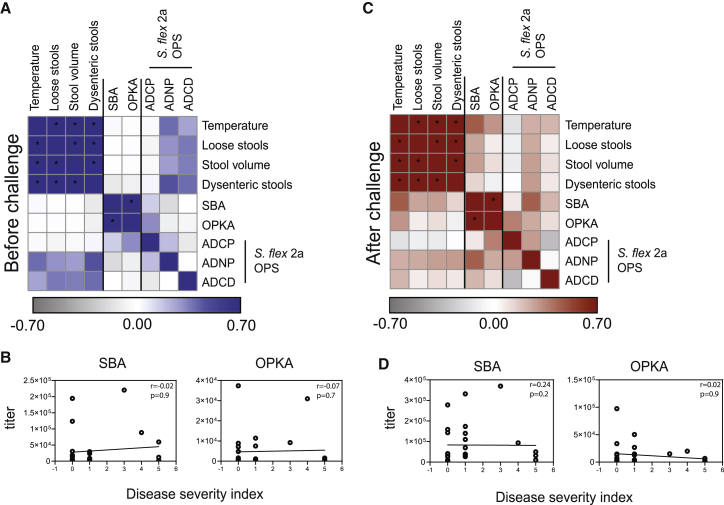
Classical SBA and OPKA assays correlate with OPS antibody functions and with shigellosis symptom severity (A and C) Heatmap of Spearman correlation of shigellosis symptoms, SBA-, OPKA-, and OPS-specific antibody-mediated functions before (A) and after (C) *S*. *flexneri* 2a challenge. (n = 27). ^∗^Corrected p < 0.05. (B and D) Spearman correlation of disease severity index with SBA or OPKA before (B) and after (D) *S*. *flexneri* 2a challenge (n = 27).

Serum bactericidal activity (SBA) and/or opsonophagocytic killing activity (OPKA) assays have been used as immune correlates of protection in several bacterial infections including meningococcal (Borrow et al., 2005), *Vibrio cholerae* (Chen et al., 2016), and *Streptococcus pneumoniae* (Romero-Steiner et al., 2006) infection. Similarly, negative associations between SBA and OPKA titers and disease have been observed against both *S*. *flexneri* 2a (Shimanovich et al., 2017) and *S*. *sonnei* (Clarkson et al., 2020). Moreover, SBA titers were recently linked to vaccine efficacy following Flexyn2a vaccination, albeit the difference in SBA across protected and unprotected vaccinees did not reach statistical significance (Clarkson et al., 2021). Therefore, we next attempted to assess the relationship between these antimicrobial functions, systems serology, and symptom severity. Prior to challenge (Figure 5A) SBA and OPKA were strongly correlated with one another (r = 0.9, p < 0.0001), modestly correlated with OPS-specific monocyte phagocytosis (r = 0.15 and r = 0.32, respectively), and poorly associated with any symptoms. Interestingly, SBA and OPKA were weakly inversely correlated with number of dysenteric stools (r = −0.1 and r = −0.08, respectively), but no other symptoms or composite severity (Figure 5B). After challenge (Figure 5C), SBA remained strongly correlated with OPKA (r = 0.8, p < 0.0001), and both SBA and OPKA correlated better with OPS-specific antibody functions. Namely, OPKA positively correlated with OPS-specific ADCP (r = 0.3, p = 0.06), both SBA and OPKA positively correlated with OPS-specific ADNP (r = 0.4, p = 0.01 and r = 0.2, p = 0.2, respectively) and OPS-specific ADCD (r = 0.2 and r = 0.1, respectively) (Figure 5C). Importantly, SBA and OPKA titers after challenge were positively correlated with symptoms (Figures 5C and 5D). Only OPS-specific ADCP was negatively associated with symptoms. Because OPS-specific ADCP and SBA/OPKA titers were weakly positively correlated, these data suggest that SBA/OPKA may capture global OPS-specific functions and that particular OPS-specific opsonophagocytic functions, for example monocyte phagocytosis, may represent a stronger proxy of immunity in this CHIM study.

### Validation in a second CHIM study

**Figure 6:**
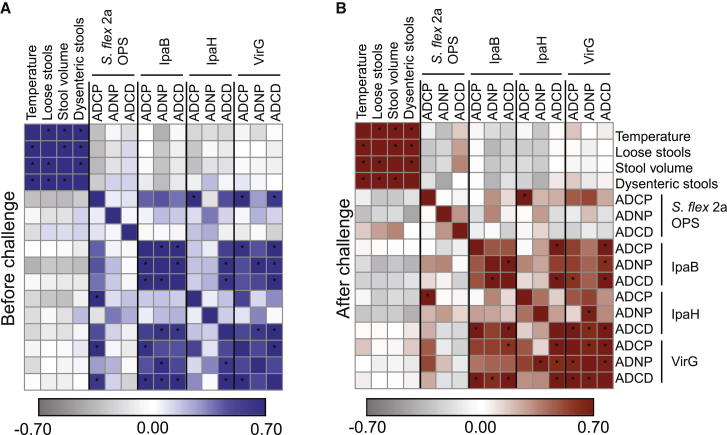
A validation challenge cohort corroborates IpaB antibody protective function (A and B) Heatmap of Spearman correlations of shigellosis symptoms, OPS-, IpaB-, IpaH-, and VirG-specific antibody-mediated functions before (A) and after (B) *S*. *flexneri* 2a challenge in a validation cohort (n = 20). ^∗^Corrected p < 0.05.

To verify our findings, we further analyzed a second independent cohort of 20 individuals (cohort 2) challenged in the same setting with *S*. *flexneri* 2a at one of two doses: 1.4 × 10^3^ CFU or 1.4 × 10^2^ CFU (Table S1) (Kotloff et al., 1995b). In the absence of significant differences in mean antigen-specific antibody titers across the two dose groups (Figure S7), all samples were analyzed together. Correlation analysis of antibody function and symptoms prior to challenge (similar to that described for cohort 1) revealed a weak positive correlation between OPS ADCD and number of loose stools, number of dysenteric stools, and stool volume (r = 0.1 for all) (Figure 6A). Conversely, OPS ADCP and IpaB ADNP negatively correlated with all shigellosis symptoms (Figure 6A), underscoring the importance of pre-existing, circulating OPS- and IpaB-specific functions for protection against shigellosis. Further comparison of OPS ADCP before challenge between the first and second cohort verified the presence of functional serum antibodies in the second cohort before challenge (Figure S8). After challenge (Figure 6B), the positive correlation of OPS-specific ADCD and shigellosis symptoms expanded (for example, r = 0.3 with number of loose stools), while IpaB ADNP and ADCD remained robustly negatively correlated with symptoms (r = −0.3 and r = −0.25 for number of loose stools, respectively). Interestingly, in this cohort VirG antibody-mediated functions before and after challenge showed minimal and inconsistent correlation with protection (Figures 6A and 6B). These findings, in an independent cohort of *Shigella* challenge, support a critical role for IpaB antibody functions in protection before and after challenge and point to a potential collaboration of these antibodies with distinct OPS-specific functional antibodies if present prior to challenge.

### Rechallenge biomarkers of protection

**Figure 7:**
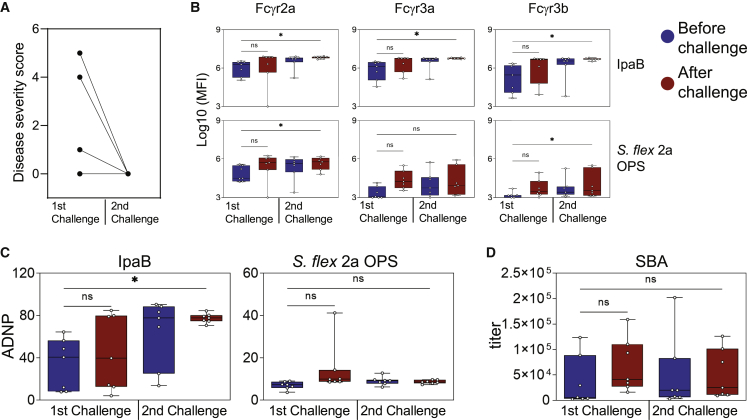
Protected individuals in a rechallenge setting harbor augmented IpaB antibody FcR binding and Fc-mediated neutrophil phagocytosis (A) Disease severity score of individuals in two consecutive challenges (n = 7). (B and C) FcR binding levels (B) and antibody-mediated neutrophil phagocytosis (C) of IpaB- and OPS-specific antibodies before and after two consecutive challenges with *S*. *flexneri* 2a (n = 7). (D) SBA titers before and after two consecutive challenges with *S*. *flexneri* 2a (n = 7). ^∗^p < 0.05; ns, not significant.

Individuals living in endemic areas and frequently exposed to *Shigella* develop natural protective immunity (Oberhelman et al., 1991; Van De Verg et al., 1992). Thus, to mimic these recurring exposures, a subgroup of seven individuals from cohort 1 (three vaccinated and four unvaccinated) were rechallenged with *S*. *flexneri* 2a 3 months after the first challenge (Table S1). In line with evidence of immunity acquired through multiple exposures, all seven rechallenged individuals had few or no shigellosis symptoms (severity score of 0) following the second challenge (Figure 7A), reaffirming the importance of pre-existing immunity in protection against disease. To determine whether particular antibody functions were selectively boosted by rechallenge, we analyzed serum samples taken before and after each challenge from seven rechallenged individuals. Both IpaB and OPS antibody FcR binding were significantly boosted following the second challenge (Figure 7B), revealing a functional maturation over time. Interestingly, IpaB ADNP boosted modestly following the first challenge but increased significantly following the second challenge (Figure 7C). Conversely, OPS-specific ADNP was moderately boosted after the first challenge but returned to low levels before the second challenge (Figure 7C), suggesting that enhanced immunity at rechallenge is not conferred by OPS-specific antibody functionality. SBA titers followed a pattern similar to that of OPS ADNP (Figure 7C). Thus, these data suggest that a functional maturation and accrual of the humoral immune response may occur selectively to particular protein antigens, such as IpaB, while OPS-specific responses may wane, both of which may contribute productively to exposure-driven enhanced immunity to *Shigella*.

## Discussion

Prevailing hypotheses have focused on OPS-specific humoral immunity as a critical correlate of immunity against *Shigella* (Cohen et al., 1991, 2019). While OPS-specific binding antibodies have been associated with reduced risk of *Shigella* dysentery in endemic settings (Cohen et al., 1988), OPS-specific antibody titers alone incompletely predict protection following vaccination; rather, the length of exposure and boosting of immunity through repeated infections more strongly associates with lower disease incidence (Passwell et al., 2010). Similarly, SBA and OPKA measurements, which largely interrogate the functional capability of OPS-specific antibodies (Ndungo et al., 2021), clearly mark the progressive acquisition of immunity, as shown in our results, but might not represent absolute predictors of protective immunity (Clarkson et al., 2021). Additional antibody specificities or qualities are evidently cooperating in protective immunity against shigellosis. However, the precise antibody specificities and functions of these antibodies, and other immune effectors and mechanisms involved, remain unknown. Such information would be invaluable to inform the development of effective vaccines as well as therapeutics against *Shigella*.

The application of systems serology, in the context of controlled experimental infection in humans, provided a unique opportunity to dissect the functional quality of the *Shigella*-specific humoral immune response associated with distinct clinical outcomes. It is important to note that our analysis was agnostic of origin of immunity and included combined serological data from challenged individuals, irrespective of previous vaccination or pre-existing immune status, stratified by clinical outcome post infection. Such an approach can identify a correlate of protection, if present, prior to challenge but cannot rule out other correlates of protection that were not detected or present in low magnitude in this sample set. A broader antibody repertoire is expected in individuals living in endemic regions and repeatedly exposed to the organism. The subjects enrolled in our studies were United States volunteers and although unexpected, some may have been exposed to *Shigella*. Hence, the markers identified may reflect pre-existing immunity to *Shigella* or cross-reactive immunity, possibly to homologous proteins in other enteric pathogens (Charles et al., 1999; Martinez-Becerra et al., 2013; Kaniga et al., 1995).

Within this context, two critical observations emerged in our analysis of samples from a non-endemic setting: pre-existing IpaB-specific IgG antibody, FcγR binding, and antibody-dependent functions were strongly linked to reduced disease severity. Similar correlations were found with VirG IgG and IpaC/VirG IgA levels and FcαR binding, but to a lesser extent. Conversely, OPS-specific humoral immune profiles were weakly associated with enhanced disease for all features except FcαR binding responses. While OPS-specific IgGs are typically considered the primary effector of *Shigella* immunity, our results uncovered the presence of subpopulations of IgA antibodies with enhanced innate immune recruiting potential as a marker of protective immunity. Intriguingly, after challenge, OPS-specific monocyte phagocytic antibodies exhibited a negative correlation with disease, raising the possibility of a synergistic effector response involving OPS-specific and IpaB antibody functions following exposure. A deeper analysis of the evolution of IpaB- and OPS-specific responses upon a consecutive challenge demonstrated a robust and conserved augmentation of functional immunity to IpaB but poorer augmentation of OPS-specific functional immunity and SBA activity, suggesting that functional immunity to OPS is limited and possibly short-lived. Collectively, these data hint to a critical collaborative role between more persistent IpaB-specific immune responses and OPS-specific functional immunity in protection against shigellosis. The low OPS-specific responses seen in the volunteers in our study are reminiscent of the low OPS seroprevalence in children younger than 2 years of age (Ndungo et al., 2021; Thompson et al., 2016), for whom the risk of shigellosis is the highest. In contrast, adults living in endemic regions and constantly exposed to *Shigella* develop stronger protein- and OPS-specific responses (Ndungo et al., 2021). The relative contribution of protein- versus OPS-specific immunity in protection against shigellosis remains a critical unanswered question.

IpaB is an essential component of the *Shigella* type III secretion system, and a major virulent factor which enables bacterial product delivery into the host cell (Pinaud et al., 2018). It is also expressed on the surface of *Shigella* and participates in epithelial cell entry (Skoudy et al., 2000; Lafont et al., 2002). While previous studies have proposed that IpaB binding antibodies could contribute to immunity by blocking IpaB activity (Heine et al., 2013; Martinez-Becerra et al., 2012), the data presented here suggest a role for IpaB antibodies in monocyte and neutrophil phagocytosis. Binding of IpaB antibodies to the Fc receptors FcγR2a and FcγR3b, which are expressed on neutrophils (Futosi et al., 2013), was linked to reduced disease severity. Given the role of neutrophils in *Shigella* clearance (Mostowy et al., 2013), this mechanism could be relevant for infection control. Hence, IgG antibodies targeting IpaB may not only block IpaB function but also drive rapid bacterial clearance after engaging this surface-expressed antigen (Skoudy et al., 2000; Lafont et al., 2002). Conversely, IgA responses following infection may contribute to blockade of the bacteria and mucosal clearance. Interestingly, IpaB is expressed across *Shigella* strains and shares similarities with homologs in other bacteria, such as *Salmonella* spp. and *Yersinia* spp. (Hueck, 1998); cross-reactivity to such homologs may account for some of the baseline protein-specific antibodies detected in our study participants.

After challenge, a significant shift in *Shigella*-specific immunity was observed, marked by expanded IgA and FcαR binding antibodies to protein antigens in protected individuals. The boost of IgA/FcαR after challenge suggests IgG-mediated early control of infection and the potential contribution of IgA in both systemic and mucosal containment and, ultimately, elimination of the pathogen. Importantly, VirG-specific ADCD was also among the post-challenge signal and was linked to a large network of IgG functions and specificities which likely strengthen IgG-mediated disease resolution, working concertedly with IgA immunity. Whether this expansion of IgA immunity is simply a marker of a mucosal immune response, a frontline antimicrobial host defense effector, or a collaborator in the IgG response to infection remains unclear but highlights the importance of future analyses of mucosal responses to gain mechanistic insights into local protective immune functions against *Shigella*. Furthermore, the expansion of OPS-specific immunity following challenge, regardless of disease severity, argues for a critical role for both IpaB- and OPS-specific immune mechanisms in the resolution of infection. Thus, while OPS immunity, which is highly serotype specific, dominates endemic immunity (Chisenga et al., 2021; Cohen et al., 1988; Van De Verg et al., 1992), the data shown here argue that vaccines able to induce a shift in natural immunodominance of the response away from OPS-only immunity to an Ipa/OPS response may drive more robust, longer-lived cross-reactive immunity. However, it will be important to explore differences in isotype and immunodominant responses in young children living in *Shigella*-endemic areas who are the most vulnerable to shigellosis and harbor an evolving immune system.

Here, using a controlled human challenge model, in which the timing and the dose of *Shigella* were tightly controlled and clinical observations post infection were carefully monitored, we uncovered a robust relationship between pre-existing IpaB functional humoral immunity with protection against shigellosis. Conversely, LPS/OPS-specific responses were weak at the time of challenge in this cohort and were poorly associated with disease. In a second challenge cohort and in the presence of a higher LPS/OPS-specific antibody baseline, both IpaB- and LPS/OPS-specific functional humoral immune responses were tied to protective immunity, suggesting that both antigenic targets contribute in a synergistic manner to enhanced protection against disease. Given the robust ability to boost IpaB-specific opsonophagocytic function, these data point out IpaB as a strong vaccine antigen candidate to complement ongoing LPS-based vaccine efforts.

### Limitations of the study

Clinical trials involving controlled human infection are complex and require special resources and infrastructure. These trials being rare, the specimens obtained are unique and highly valuable. In this study we examined samples from a CHIM study conducted in the 1990s. Specimens were stored under monitored conditions, and adequate quality was confirmed prior to experimental analysis. The participants examined included both naive individuals and individuals previously vaccinated with a hybrid EcSf2a-2 strain. The vaccine was not protective and induced limited to no immunogenicity, as was confirmed by univariate and multivariate analysis of both pre-challenge and post-challenge data. Hence, the pre-existing immune profiles were nearly identical across the groups. There was also no evidence of robust immune memory to *Shigella* in the vaccinated group that could account for differences in response to challenge. Additionally, the features that were selected by the model that resolved individuals who resisted disease were not differentially enriched across the vaccinees or naive subjects. While comparison of only truly naive individuals would have been a viable option, we opted for combined group analyses to increase the statistical power to detect differences; the similar immune profiles provided strong rationale that these individuals were comparable, combinable, and ultimately provided a signature that was confirmed in a second fully naive, albeit smaller cohort. Nonetheless, we were able to identify differences in antigen-specific responses in challenged volunteers based on disease severity. Importantly, the critical role for IpaB-specific antibody functions detected before challenge in clinical protection was corroborated using samples from cohort 2, which included all naive subjects. Future systems serology studies with larger sample sizes and especially including individuals from *Shigella*-endemic regions will help confirm our observations. Finally, our study was focused on circulating (serum) as opposed to mucosal antibodies. While serum markers may provide critical insights into the immunologic mechanisms involved in resolution of disease, mucosal profiling may provide more insight toward identifying functional mechanisms that limit microbial colonization and infection locally.

## STAR★Methods

### Key resources table

**Table:** 

REAGENT or RESOURCE	SOURCE	IDENTIFIER
**Antibodies**		

Mouse Anti-Human IgG1-Fc PE	Southern Biotech	CAT# 9054-09; RRID: AB_2796628
Mouse Anti-Human IgG2-Fc PE	Southern Biotech	CAT# 9060-09; RRID: AB_2796635
Mouse Anti-Human IgG3-Fc PE	Southern Biotech	CAT# 9210-09; RRID: AB_2796701
Mouse Anti-Human IgM-Fc PE	Southern Biotech	CAT# 9020-09; RRID: AB_2796577
Mouse Anti-Human IgA1-Fc PE	Southern Biotech	CAT# 9130-09; RRID: AB_2796656
Mouse Anti-Human IgA2-Fc PE	Southern Biotech	CAT# 9140-09; RRID: AB_2796664
Pacific Blue(TM) anti-human CD66b antibody	Biolegend	CAT# 305112; RRID: AB_2563294

**Chemicals**, **peptides**, **and recombinant proteins**		

*Shigella* recombinant proteins: IpaB, IpaC, IpaD, VirG and *Shigella* LPS	N/A	Provided by WRAIR
*Shigella* IpaH		Provided by Vaxcyte
BSA conjugated *Shigella flexneri* 2a O-polysaccharide	N/A	Isolated and produced at the Ryan lab/MGH, conjugated to BSA at NIH
Zaire ebolavirus glycoprotein	R&D Systems	CAT#9016-EB
Human Fc receptors	Produced at the Duke Human Vaccine Institute, {Boesch, 2014 #15}	N/A
Streptavidin-R-Phycoerythrin	Prozyme	CAT# PJ31S
EDC (1-ethyl-3-(3-dimethylaminopropyl)carbodiimide hydrochloride)	Thermo Fisher	CAT# 77,149
Sulfo-NHS-LC-LC biotin	Thermo Fisher	CAT# A35358
DMTMM (4-(4,6-Dimethoxy-1,3,5-triazin-2-yl)-4-methylmorpholinium chloride)	Sigma Aldrich	74,104
Lyophilized guinea pig complement	Cedarlane	CL4051

**Software and algorithms**		

IntelliCyt ForeCyt (v8.1)	Sartorius	https://intellicyt.com/products/software/
FlowJo (v10.7.1)	FlowJo, LLC	https://www.flowjo.com/solutions/flowjo
Prism 9.2.0 (283)	GraphPad	https://www.graphpad.com/scientific-software/prism/

**Other**		

FluoSpheres™ NeutrAvidin™-Labeled Microspheres, 1.0 μm, yellow-green fluorescent (505/515), 1% solids	Invitrogen	CAT# F8776
FluoSpheres™ Carboxylate-Modified Microspheres, 1.0 μm, blue (fluorescent 350/440), 1% solids	Invitrogen	CAT# F8815
FluoSpheres™ NeutrAvidin™-Labeled Microspheres, 1.0 μm, crimson fluorescent (625/645),1% solids	Invitrogen	
FluoSpheres™ NeutrAvidin™-Labeled Microspheres, 1.0 μm, red-orange fluorescent (565/580)1% solids	Invitrogen	
MagPlex microspheres	Luminex corporation	CAT# MC12001-01

### Resource availability

#### Lead contact

Further information and requests for resources and reagents should be directed to and will be fulfilled by the lead contact, Galit Alter (GALTER@mgh.harvard.edu).

#### Materials availability

This study did not generate new unique reagents.

### Experimental model and subject details

#### Clinical studies and human serum samples

Serum samples were obtained from clinical studies performed on healthy community volunteers at the Center for Vaccine Development (University of Maryland, Baltimore) under approved IRB protocols: (i) Cohort 1: Phase IIb *Shigella* challenge study that included 16 adult volunteers orally immunized with a hybrid *E*. *coli*- *Shigella* LPS EcSf2a-2 vaccine strain and 11 unvaccinated controls. One month after the last vaccine dose, these individuals were challenged with 1x10^3^ CFU of wild-type (WT) strain *S*. *flexneri* 2a 2457T. Details of the vaccination and challenge procedures are described elsewhere (Kotloff et al., 1995a). Specimens obtained at days −1 (prior to challenge) and 28 (post challenge) from all 16 vaccinated and 11 unvaccinated individuals were analyzed (Table S1). Eleven of these individuals were then selected to participate in a homologous re-challenge trial (1.4x10^3^ CFU of the *S*. *flexneri* 2a WT strain) assessing protective immunity conferred by previous shigellosis (Kotloff et al., 1995b); serum from 7 individuals (3 vaccinated and 4 naive) were evaluated in our study. (ii) Cohort 2: In addition, 13 immunologically naive subjects (who never received the hybrid vaccine or challenge strain) were recruited and challenged with 1.4x10^3^ CFU of the *S*. *flexneri* 2a WT strain. Seven additional volunteers were inoculated with a lower dose (1.4x10^2^ CFU) (Table S1). Details of the re-challenge study procedures were described previously (Kotloff et al., 1995b). Serum from all 20 immunologically naive individuals were evaluated in our study (Table S1). Clinical samples were obtained using established procedures and properly stored in dedicated freezers (CVD clinical repository). Gender information of study participants is unknown.

A categorical 4-point disease index was previously used to score disease outcomes (Shimanovich et al., 2017). An additional disease severity based on symptoms and signs of disease including body temperature, stool consistency, number of stools and dysenteric stools was calculated as previously described (Porter et al., 2018). Disease outcomes were scored separately and then combined to calculate DSI. Raw numbers for each study participant are available (Figure S4).

#### Primary immune cells

Fresh peripheral blood was collected by the MGH Blood bank from healthy human volunteers. All volunteers gave signed consent and were over 18 years of age, and all samples were de-identified before use. The study was approved by the MGH Institutional Review Board. Human neutrophils were isolated from fresh peripheral blood and maintained at 37°C, 5% CO_2_ in RPMI with 10% fetal bovine serum, L-glutamine, penicillin/streptomycin.

#### Cell lines

THP-1 cells (ATCC), a monocytic leukemia cell line, were maintained in RPMI supplemented with 10% fetal bovine serum, L-glutamine, penicillin/streptomycin, HEPES, and beta-mercaptoethanol. THP-1 cells were grown at 37°C, 5% CO_2_.

### Method details

#### Luminex

*Shigella*-specific antibody subclass/isotype and Fcγ-receptor (FcγR) binding levels were assessed using a 384-well based customized multiplexed Luminex assay, as previously described (Brown et al., 2018). IpaB (purchased from Walter Reed Army Institute of Research, WRAIR), IpaC (WRAIR), IpaD (WRAIR), IpaH (Vaxcyte), VirG (WRAIR), *S*. *flexneri* 2a 2457T LPS (WRAIR), *S*. *sonnei* Moseley LPS (WRAIR), and *S*. *flexneri* 2a OPS were used to profile specific humoral immune response. OPS was purified from *S*. *flexneri* 2a SFX2a001 LPS by acid hydrolysis and size exclusion chromatography and conjugated to BSA. Tetanus toxin and Zair Ebola glycoprotein (CEFTA, Mabtech Inc, R&D systems) were used as a control. Protein antigens were coupled to magnetic Luminex beads (Luminex Corp) by carbodiimide-NHS ester-coupling (Thermo Fisher). OPS and LPS antigens were modified by 4-(4,6-dimethoxy[1,3,5]triazin-2-yl)-4-methyl-morpholinium and conjugated to Luminex Magplex carboxylated beads. Antigen-coupled microspheres were washed and incubated with serum samples at an appropriate sample dilution (1:50 for Isotypes and 1:100 for all Fc-receptors) for 2 h at 37°C in 384-well plates (Greiner Bio-One). Unbound antibodies were washed away, and antigen-bound antibodies were detected by using a PE-coupled detection antibody for each subclass and isotype (IgG1, IgG2, IgG3, IgA1, IgA2 and IgM; Southern Biotech), and Fcγ-receptors were fluorescently labeled with PE before addition to immune complexes (FcγR2A, FcγR2B, FcγR3A, FcγR3B, FcαR; Duke Protein Production facility). After 1 h incubation, plates were washed, flow cytometry was performed with an IQue (Intellicyt), and analysis was performed on IntelliCyt ForeCyt (v8.1). PE median fluorescent intensity (MFI) was reported as a readout for antigen-specific antibody titers.

#### Antibody-dependent monocyte and neutrophil phagocytosis (ADCP and ADNP)

ADCP and ADNP were conducted as previously described (Butler et al., 2019). IpaB, IpaH and VirG were biotinylated using Sulfo-NHS-LC-LC biotin (Thermo Fisher) and coupled to fluorescent Neutravidin-conjugated beads (Thermo Fisher). *S*. *flexneri* 2a OPS was modified with DMTMM and coupled to carboxylated fluorescent beads (Thermo Fisher). To form immune complexes, a mix of four antigen-coupled beads was incubated for 2 h at 37°C with diluted samples (1:50 for ADCP, 1:25 for ADNP) and then washed to remove unbound immunoglobulins. For ADCP, the immune complexes were incubated for 16–18 h with THP-1 cells (1.25x10^5^ cells/mL) and for 1 h with RBC-lyzed whole blood for ADNP. Following the incubation, cells were fixed with 4% PFA. For ADNP, RBC-lyzed whole blood was washed, stained for CD66b (Biolegend) to identify neutrophils, and then fixed in 4% PFA. Flow cytometry was performed to identify the percentage of cells that had phagocytosed beads as well as the number of beads that had been phagocytosed (phagocytosis score = % positive cells × Median Fluorescent Intensity of positive cells/10000). The Flow cytometry was performed with an LSRII(BD), and analysis was performed using FlowJo V10.7.1.

#### Antibody dependent complement deposition (ADCD)

ADCD was conducted using a 384-well based customized multiplexed assay. Protein antigens were coupled to magnetic Luminex beads (Luminex Corp) by carbodiimide-NHS ester-coupling (Thermo Fisher). OPS and LPS antigens were modified by 4-(4,6-dimethoxy[1,3,5]triazin-2-yl)-4-methyl-morpholinium and conjugated to Luminex Magplex carboxylated beads Schlottmann et al., 2006). To form immune complexes, a mix of four antigen-coupled beads was incubated for 2 h at 37°C with diluted samples (1:10) and then washed to remove unbound immunoglobulins. Lyophilized guinea pig complement (Cedarlane) was resuspended according to manufacturer’s instructions and diluted in gelatin veronal buffer with calcium and magnesium (Boston BioProducts). Resuspend guinea pig complement was added to immune complexes and incubated for 20 min at 37°C. Post incubation, C3 was detected with Fluorescein-Conjugated Goat IgG Fraction to Guinea Pig Complement C3 (Mpbio).

#### SBA and OPKA assays

SBA and OPKA assays were performed as previously described (Ndungo et al., 2021). Titers were determined as the reciprocal of the serum dilution that produced 50% bacterial killing. The provisional reference serum sample, Korean QC19 (assigned titer = 28,000) (Nahm et al., 2018), was used to normalize SBA titers.

### Quantification and statistical analysis

#### Data pre-processing

The raw MFI was scaled by the log10 function and was then subtracted by the corresponding PBS values. The normalized MFI values were assigned to zero if they were negative.

#### Statistics

Microsoft Excel 365 was used to compile experimental data and disease information. Violin plots, bar graphs, and x-y plots were generated in Graph Pad Prism V.8. Statistical differences between two groups were calculated using a two-sided Mann–Whitney test or Wilcoxon test for paired comparisons. To compare multiple groups, a Kruskal–Wallis test was used followed by the Dunn’s method correcting for multiple comparisons in Graph Pad Prism V.8 (significance levels: ^∗^p < 0.05, ^∗∗^p < 0.01, ^∗∗∗^p < 0.001, ^∗∗∗∗^p ≤ 0.0001). Statistical details can be found in the Results section and in figure legends. Heat maps and correlation matrix were created using Morpheus (https://software.broadinstitute.org/morpheus) or R version 1.4.1106.

#### Computational analysis

PCA analysis was carried out using R prcomp function (Wold et al., 1987). A supervised multivariate analysis method of Least Absolute Shrinkage and Selection Operator (LASSO) followed by Partial Least-Squares Regression (PLSR) was used to identify key antibody features that contribute to variation in the disease severity. Prior to building the LASSO-PLSR model, all titer, FcR and ADCD measurements were log transformed, and all measurements were then z-scored. LASSO identified a minimal set of features that drives separation in samples of varying disease severity. LASSO selected features were used to build the PLSR model regressing against the disease severity score. The performance of the algorithm was evaluated with R^2^ and Q^2^ metrics. For “before” model, R^2^ was 0.75 indicating a high predictive accuracy and Q^2^, which indicates performance on test data in the cross-validation setting was 0.57. R^2^ and Q^2^ were 0.67 and 0.59 respectively for “after” model. Features were ranked based on their Variable of Importance (VIP) score and the loadings of the latent variable 1 (LV1) was visualized in a bar graph, which captures the contribution of each feature to the variation in disease severity. These analyses were carried out using R package *glmnet* (v4.0.2) (Friedman et al., 2010) and *ropls* (v1.20.0) (Thévenot et al., 2015). Co-correlate networks were constructed based on the pairwise correlation between the top predictive features selected for disease severity and all measured biophysical and functional features. Only correlations with an absolute Spearman correlation coefficient greater than 0.7 and p value lower than 0.01 after correction for multiple comparisons by Benjamini-Hochberg (BH) were shown. Networks were generated using R package *network* (v1.16.0) [Butts C (2015). network: Classes for Relational Data.].

## Data Availability

The dataset generated during and/or analyzed during the current study have been made available in the supplemental material. This paper does not report original code. Any additional information required to reanalyze the data reported in this work paper is available from the lead contact upon request.

## Supplemental information

Document S1. Figures S1–S8

Table S1. Clinical samples used in study

Data S1. AllCHIMBeforeData

Document S2. Article plus supplemental information
