# Supplementary material for: Systems approach to define humoral correlates of immunity to Shigella
Source: Cell Rep. 2022 Aug 16;40(7):111216. doi: 10.1016/j.celrep.2022.111216 (PMC9396529; doi:10.1016/j.celrep.2022.111216)
Supplement: Document S1. Figures S1–S8 [file mmc1.pdf]

**Cell Reports, Volume *40***

## Supplemental information

# Systems approach to define humoral correlates of immunity to *Shigella*

**Biana Bernshtein, Esther Ndungo, Deniz Cizmeci, Peng Xu, Pavol Kováč, Meagan Kelly, Dilara Islam, Edward T. Ryan, Karen L. Kotloff, Marcela F. Pasetti, and Galit Alter**

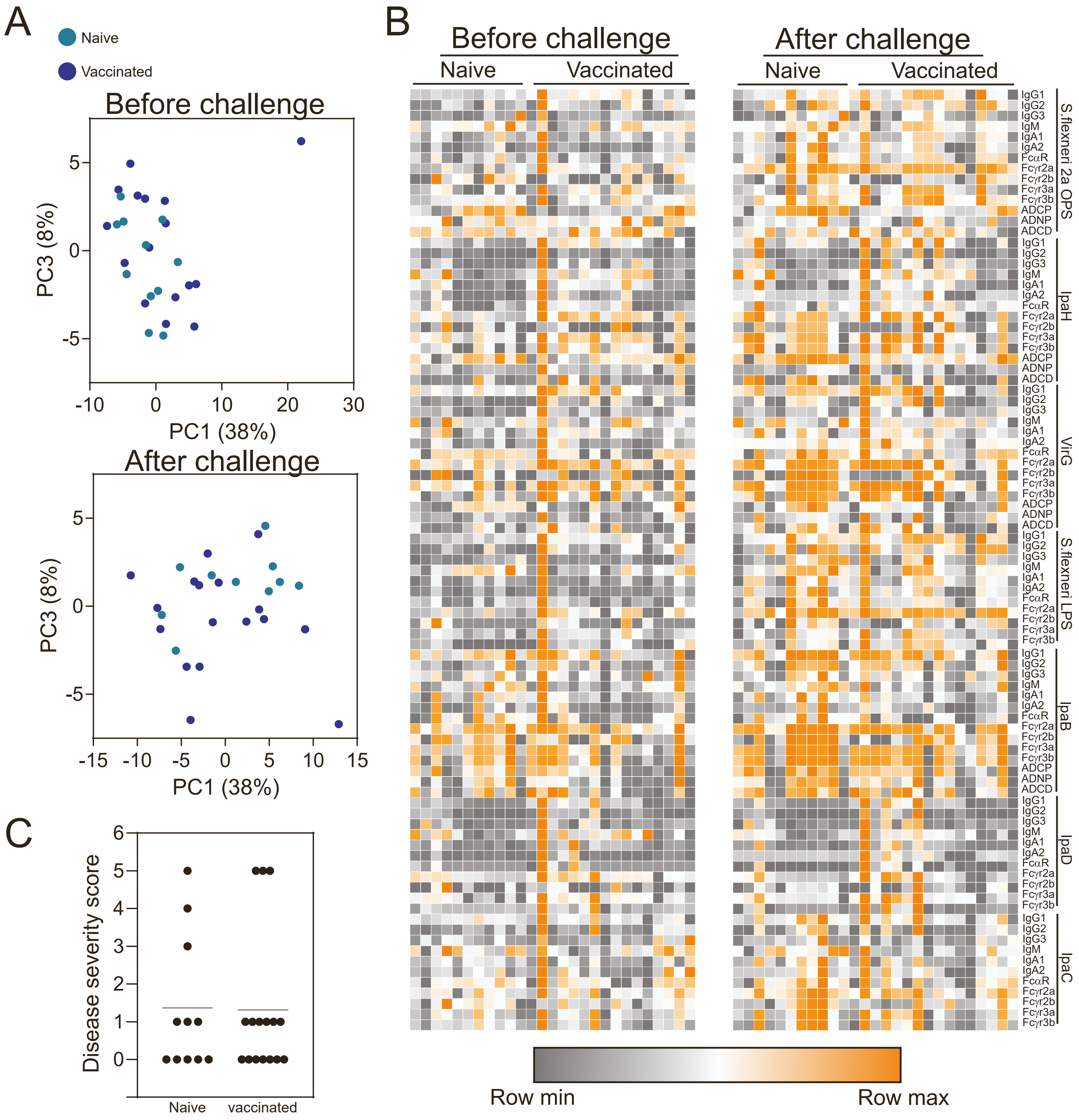


SuppFig1. Related to Fig1 (A) PCA of antibody profiles before and after S. flexneri 2a challenge of vaccinated and naïve study participants (B) Heat map depicting antibody isotype and FcR binding before and after challenge with S. flexneri 2a of vaccinated and naïve study participants (C)Disease severity score of vaccinated and naïve individuals

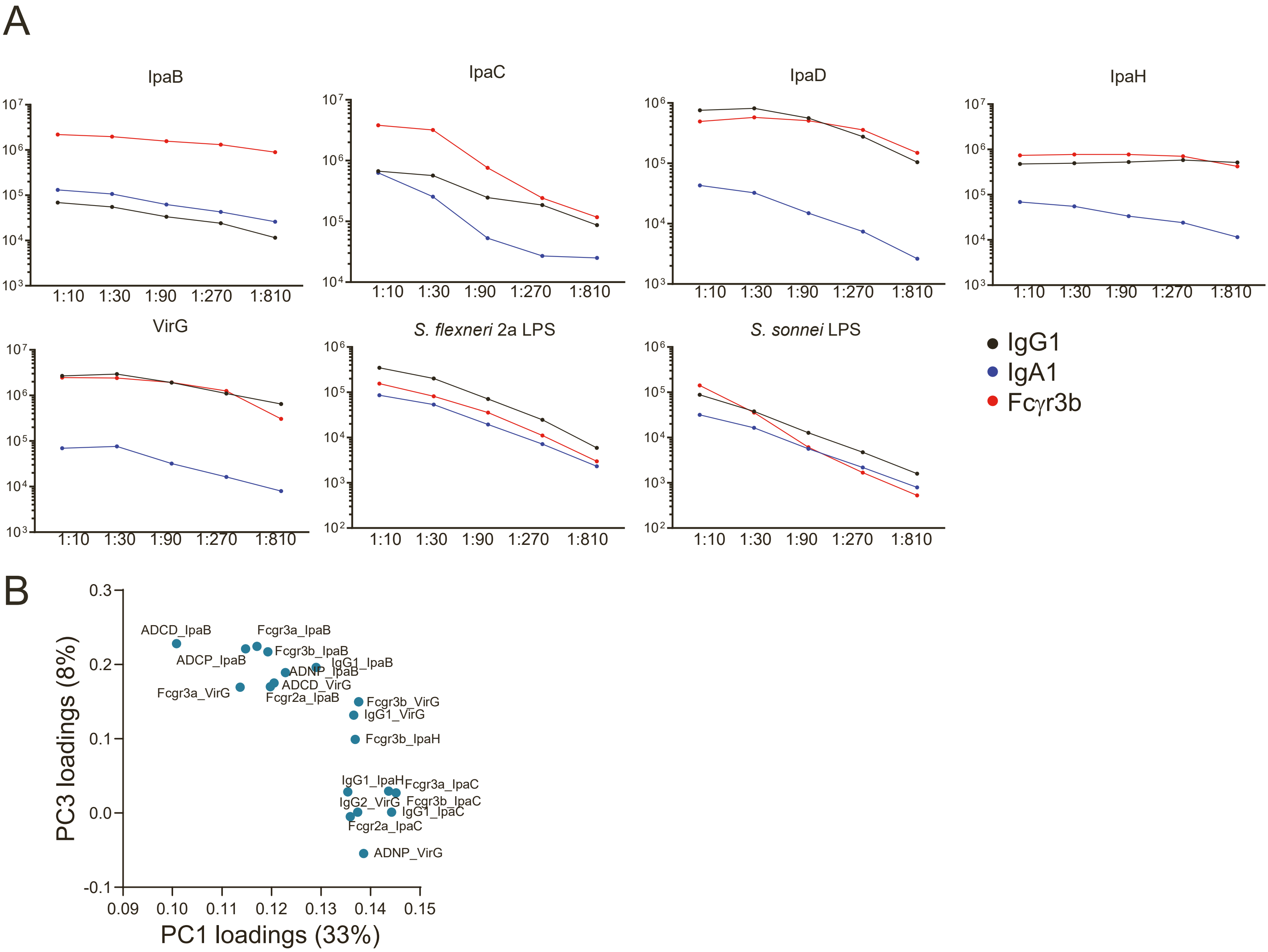


Supp Fig2. Related to Fig1. (A) Dilution curves of Shigella antigen-specific antibody binding by Luminex. (B) PC1 and PC3 loadings of PCA plot presented in Fig 1B

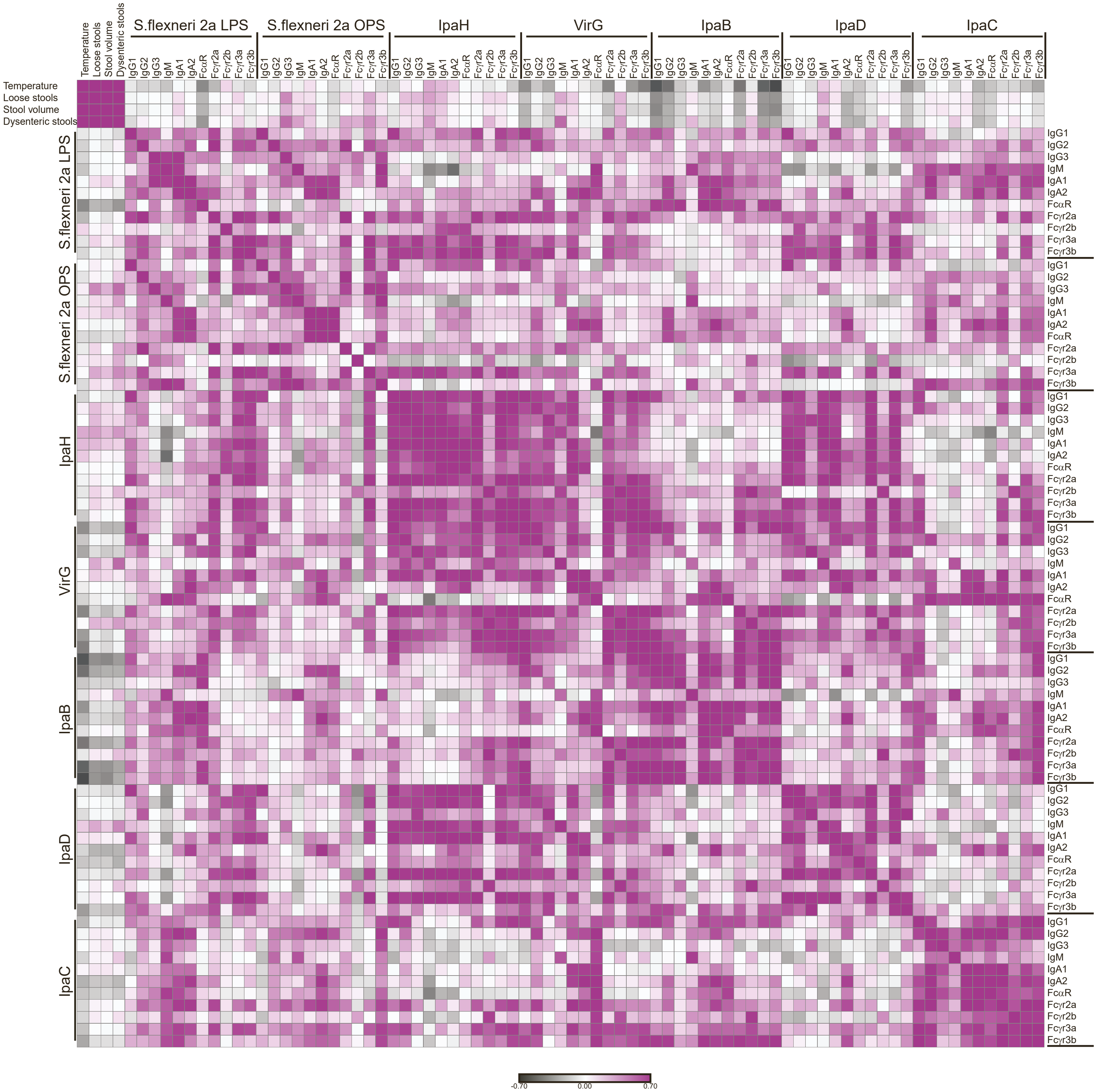


SuppFig3. Related to Fig2. Heat map of Spearman correlation between shigellosis symptoms and Shigella antigen specific antibody profiles before S. flexneri 2a challenge.

| 0-3 disease score | disease severity index | Temperature (F) | Loose stools | Stool volume | Dysenteric stools |
|---|---|---|---|---|---|
| 0 | 0 | 98.7 | 0 | 0 | 0 |
| 0 | 0 | 98.7 | 0 | 0 | 0 |
| 0 | 0 | 98.7 | 0 | 0 | 0 |
| 0 | 0 | 98.7 | 0 | 0 | 0 |
| 0 | 0 | 98.7 | 0 | 0 | 0 |
| 1 | 1 | 101.7 | 3 | 459 | 0 |
| 1 | 2 | 98.7 | 8 | 936 | 0 |
| 1 | 0 | 98.7 | 3 | 140 | 1 |
| 2 | 4 | 103 | 16 | 1982 | 5 |
| 2 | 2 | 101.4 | 7 | 1030 | 6 |
| 2 | 4 | 103.8 | 10 | 1058 | 8 |
| 2 | 1 | 102.9 | 3 | 281 | 0 |
| 2 | 3 | 102.4 | 16 | 2753 | 2 |
| 2 | 1 | 101.5 | 9 | 1390 | 1 |
| 2 | 1 | 98.7 | 4 | 366 | 3 |
| 2 | 2 | 102 | 9 | 680 | 7 |
| 3 | 2 | 104.2 | 15 | 3828 | 6 |
| 3 | 4 | 103.9 | 14 | 2268 | 5 |
| 3 | 5 | 103.5 | 26 | 4315 | 18 |
| 3 | 2 | 104.1 | 13 | 1269 | 8 |
| 0 | 0 | 99.2 | 0 | 0 | 0 |
| 0 | 0 | 99.1 | 0 | 0 | 0 |
| 0 | 0 | 98.9 | 0 | 0 | 0 |
| 0 | 0 | 98.3 | 0 | 0 | 0 |
| 1 | 1 | 99.7 | 4 | 113 | 1 |
| 1 | 0 | 99.2 | 1 | 13 | 0 |
| 1 | 0 | 100.8 | 3 | 174 | 0 |
| 1 | 1 | 101.5 | 1 | 114 | 0 |
| 2 | 1 | 101.2 | 5 | 374 | 2 |
| 2 | 1 | 103.2 | 2 | 180 | 1 |
| 2 | 1 | 100.5 | 9 | 276 | 7 |
| 2 | 3 | 102.8 | 8 | 1484 | 2 |
| 3 | 5 | 103.2 | 34 | 2381 | 23 |
| 3 | 5 | 104.3 | 35 | 2249 | 32 |
| 3 | 5 | 102.9 | 32 | 1923 | 14 |
| 3 | 5 | 103 | 29 | 1574 | 21 |
| 2 | 1 | 102.3 | 5 | 574 | 1 |
| 0 | 0 | 98.9 | 0 | 0 | 0 |
| 1 | 1 | 101.3 | 0 | 0 | 0 |
| 1 | 0 | 99.3 | 3 | 253 | 0 |
| 2 | 1 | 102.4 | 7 | 368 | 4 |
| 1 | 0 | 100.5 | 2 | 22 | 2 |
| 2 | 1 | 103.9 | 11 | 2065 | 2 |
| 2 | 4 | 102.5 | 14 | 974 | 7 |
| 0 | 0 | 98.7 | 0 | 0 | 0 |
| 1 | 0 | 98.7 | 3 | 217 | 2 |
| 0 | 0 | 98.2 | 0 | 0 | 0 |
| 0 | 0 | 98.7 | 0 | 0 | 0 |
| 0 | 0 | 98.7 | 0 | 0 | 0 |
| 0 | 0 | 98.7 | 0 | 0 | 0 |
| 0 | 0 | 98.7 | 0 | 0 | 0 |
| 0 | 0 | 98.7 | 0 | 0 | 0 |
| 1 | 0 | 98.7 | 3 | 100 | 0 |
| 1 | 0 | 100.5 | 2 | 183 | 0 |

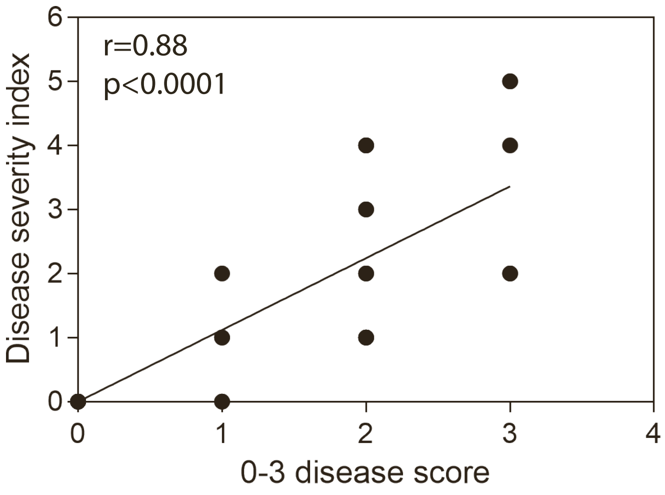


SuppFig4. Related to Figures 2,3,4,5 and 7. Table of measured shigellosis symptoms and disease severity scores and spearman correlation between 0-3 disease scores and disease severity index used in this manuscript.

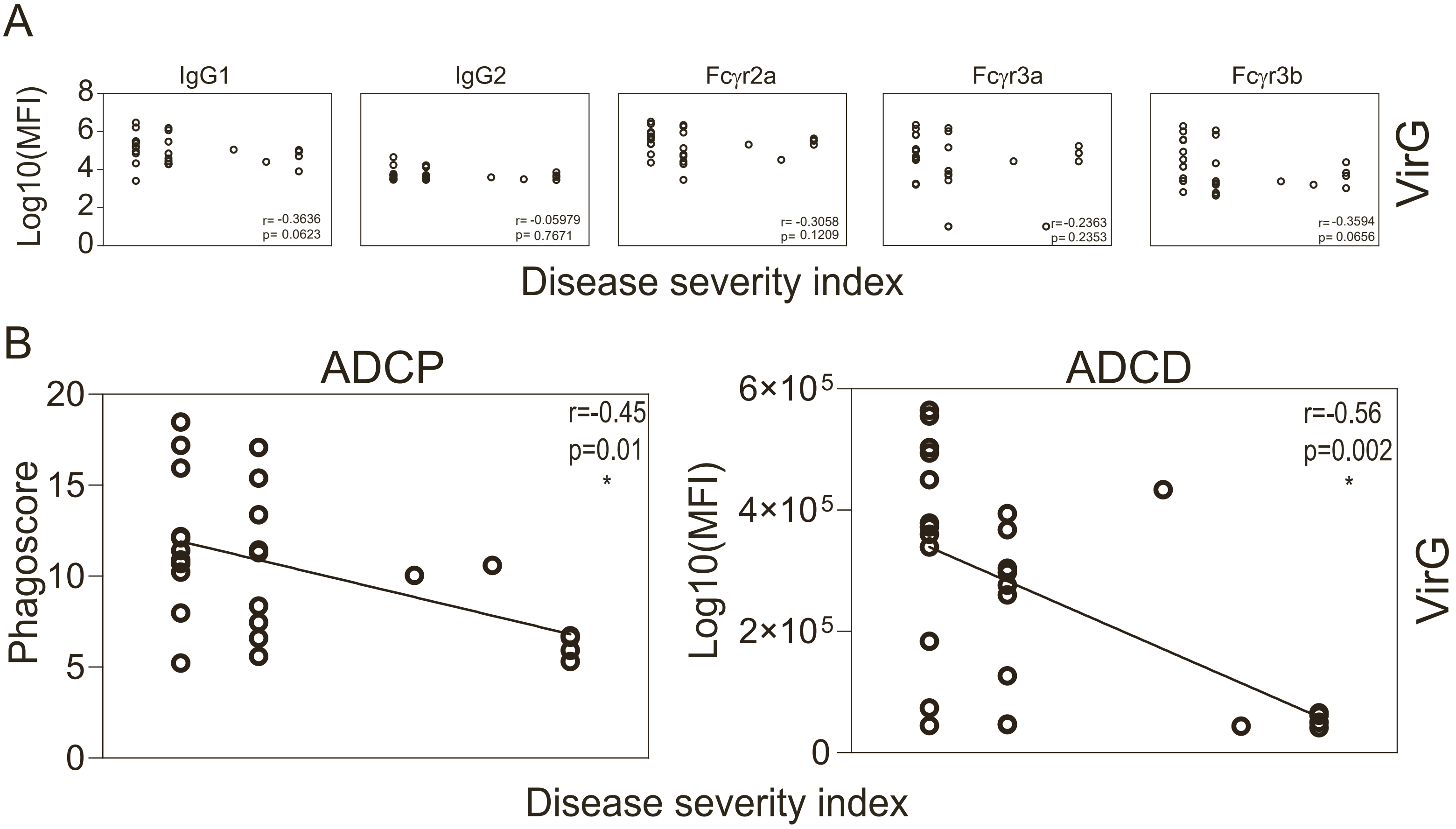


SuppFig5. Related to Figures 2 and 3. Spearman correlation of disease severity index and VirG-specific antibody-mediated functions before S. flexneri 2a challenge.

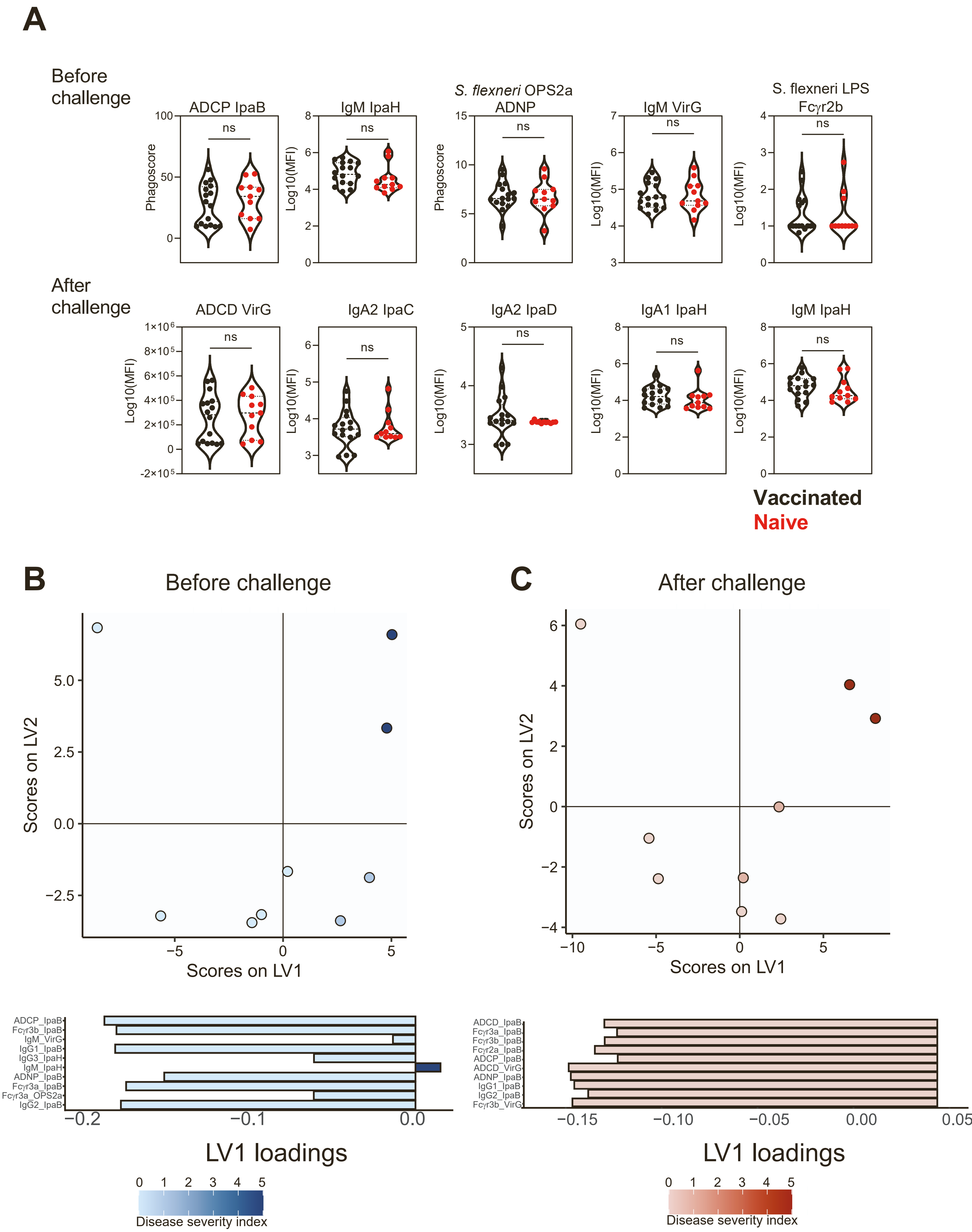


SuppFig6. Related to Figure 4. (A) LASSO selected antibody features from PLSR in Fig4 before and after S.flexneri 2a challenge of vaccinated and naïve study participants. (B-C) PLSR and top 10 VIP antibody features before (B) and after (C) challenge in naïve individuals from CHIM1 (n=11).

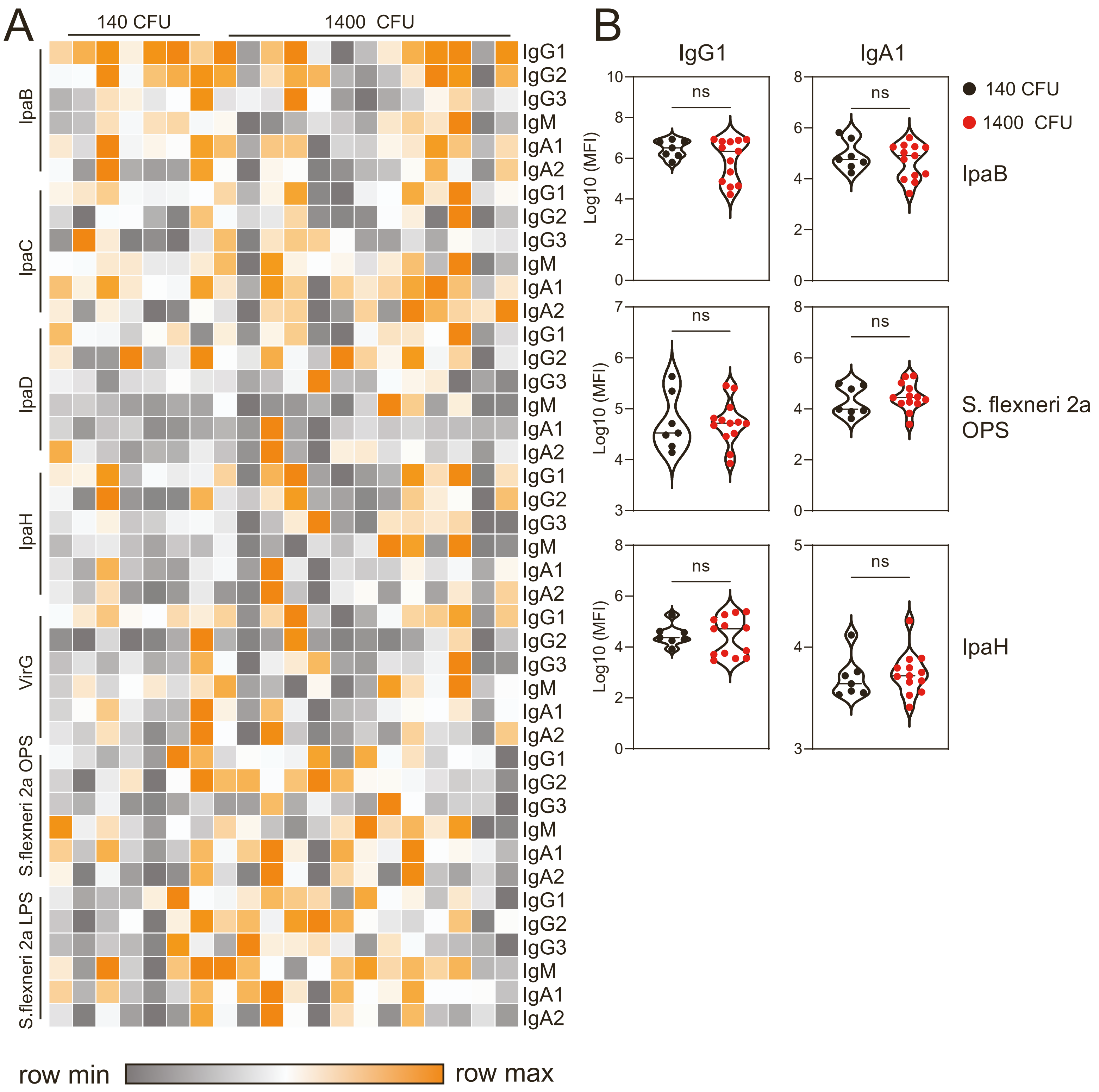


SuppFig7. Related to Figure 6. (A) Heat map depicting antibody isotype after challenge with S. flexneri 2a in high dose (1400 CFU) and low dose (140 CFU) recipients. (B) IgG1 and IgA1 levels of IpaB, S. flexneri 2a OPS and IpaH specific antibodies after S. flexneri 2a challenge in high dose (1400 CFU) and low dose (140 CFU) recipients measured by Luminex (wilcoxon test).

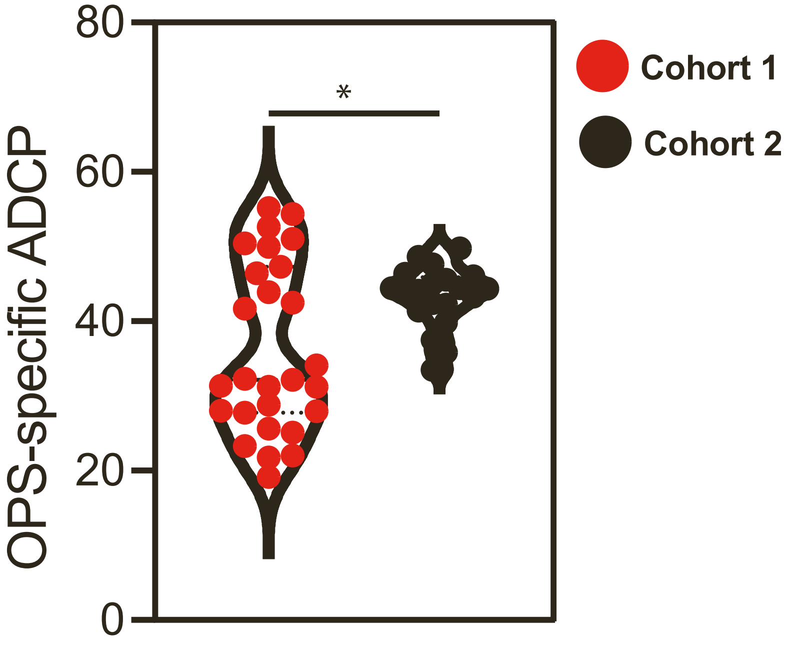


SuppFig8. Related to Figure 6. OPS-specific antibody-mediated phagocytosis before S. flexneri 2a challenge in first cohort and validation cohort (*P<0.05, wilcoxon test).
