# Supplementary material for: Systems approach to define humoral correlates of immunity to Shigella
Source: Cell Rep. 2022 Aug 16;40(7):111216. doi: 10.1016/j.celrep.2022.111216 (PMC9396529; doi:10.1016/j.celrep.2022.111216)
Supplement: Table S1. Clinical samples used in study [file mmc2.docx]

| **Supp Table 1. Clinical samples used in study** | | | | |
| --- | --- | --- | --- | --- |
| Group | No. of subjects | Challenge inoculum 1 | No. of subjects | Challenge inoculum 2 |
| Cohort 1 | 27 | 1 x 10^3^ CFU | 7 | 1.4 x 10^3^ CFU |
| Cohort 2 | 13 | 1.4 x 10^3^ CFU |  |  |
|  | 7 | 1.4 x 10^2^ CFU |  |  |
